# Comparison of the Effectiveness of Tamoxifen and Mitomycin C in the Prevention of Recurrent Urethral Stricture Following Internal Optical Urethrotomy (IOU): A Randomized Controlled Trial

**DOI:** 10.7759/cureus.94384

**Published:** 2025-10-12

**Authors:** Saifullah Khan, Faiza Hayat, Qudratullah Wazir, Sara Kalsomm, Maheen Zahid

**Affiliations:** 1 Department of Urology, Institute of Kidney Diseases, Hayatabad Medical Complex, Peshawar, PAK; 2 Department of Urology, King Edward Medical University, Lahore, PAK

**Keywords:** internal optical urethrotomy, mitomycin c (mmc), recurrence, tamoxifen, urethral stricture

## Abstract

Internal optical urethrotomy (IOU) is a common surgical procedure for treating anterior urethral strictures, but recurrence remains a significant concern. The aim of the study is to compare the effectiveness of oral tamoxifen versus mitomycin C (MMC) in preventing recurrence and improving functional outcomes following IOU for anterior urethral stricture.

This prospective, randomized controlled trial enrolled 60 male patients with anterior urethral strictures ≤2 cm in length. Participants were randomized 1:1 to receive oral tamoxifen or MMC immediately following IOU. Outcomes assessed at baseline, three months, and six months included the International Prostate Symptom Score (IPSS) as the primary outcome, and secondary measures, including maximum urinary flow rate (Qmax), stricture morphology on retrograde urethrogram, recurrence rate, and recurrence-free period. Recurrence-free period was analyzed separately using Kaplan-Meier analysis. Statistical analysis used the independent-samples t-test, Chi-square test, and Kaplan-Meier analysis, with p <0.05 considered significant.

Baseline characteristics were similar between groups. At three months, both groups showed significant improvement in IPSS and Qmax, with no intergroup difference. At six months, tamoxifen patients had significantly lower IPSS (12.33 ± 2.29 vs. 19.23 ± 3.66; p < 0.001), higher Qmax (11.22 ± 1.14 vs. 7.77 ± 0.91 mL/s; p < 0.001), and narrower stricture width (1.76 ± 0.28 vs. 3.07 ± 0.38 mm; p < 0.001) than MMC patients. Recurrence occurred in 23.3% of tamoxifen and 20.0% of MMC patients (p = 0.787). Kaplan-Meier analysis showed no significant difference in recurrence-free period (p > 0.05).

Both tamoxifen and MMC improved urinary function after IOU, but tamoxifen provided superior functional improvement at six months, despite similar recurrence rates. These findings support further evaluation of tamoxifen as an adjuvant to IOU in larger, long-term trials.

## Introduction

Urethral stricture disease is a prevalent and challenging urologic condition characterized by fibrotic narrowing of the urethra [1], resulting in obstructive urinary symptoms, recurrent infections, and impaired quality of life. It is estimated to account for more than 1.5 million urology visits annually in the United States, with associated costs exceeding $6,500 per insured male [2]. The prevalence rises with age - from approximately 200 per 100,000 men in younger cohorts to over 900 per 100,000 in 70-year-old men [3]. The majority of strictures involve the anterior urethra, particularly the bulbar segment, with average stricture lengths around 2-4 cm [4]. Despite advances in surgical techniques, urethral stricture continues to represent a substantial health and economic burden worldwide.

The pathogenesis of urethral stricture involves a cascade of maladaptive wound healing, leading to spongiofibrosis. Trauma, infection, or iatrogenic injury disrupts the urethral epithelium, often resulting in urine extravasation and subsequent fibro-inflammatory changes in the corpus spongiosum [5]. Histopathological studies show replacement of normal connective tissue with dense collagen bundles, a reduced type III-to-I collagen ratio, and diminished smooth muscle content - compromising tissue elasticity and urethral patency [6]. At the molecular level, TGF-β1 has been identified as a central profibrotic mediator, increasing collagen synthesis and deposition in urethral tissue [7]. Recent studies have also implicated microRNAs (e.g., miR-129-5p, miR-135a-5p) in regulating TGF-β-mediated fibrogenesis in pelvic trauma-associated strictures [8]. Matrix stiffness has emerged as a crucial biomechanical driver of fibrosis. In urethral stricture disease, scar tissue exhibits markedly increased stiffness compared to normal urethra, which promotes fibroblast-to-myofibroblast transition via the Igfbp3/Smad2/3 axis [9]. More broadly, mechanotransduction through integrins, Rho-ROCK signaling, and YAP/TAZ nuclear activation is well established in other fibrotic contexts, perpetuating myofibroblast activation and extracellular matrix deposition [10]. Together, these insights underscore fibrosis as the central target for therapeutic intervention.

Internal optical urethrotomy (IOU), also known as direct visual internal urethrotomy (DVIU), remains the most commonly performed first-line surgical treatment due to its minimally invasive nature and short recovery time. However, it suffers from poor long-term durability, with recurrence rates reported between 20% and 60% within 6-12 months, and even higher over extended follow-up [11]. Consequently, there is growing interest in adjunctive antifibrotic therapies aimed at reducing recurrence rates post-IOU.

Mitomycin C (MMC), an antineoplastic agent with potent antifibrotic properties, has been extensively studied as an intralesional adjunct. Multiple randomized controlled trials and meta-analyses have demonstrated that MMC significantly reduces recurrence rates following IOU, with pooled odds ratios of approximately 0.27 [12] and 0.41 [13] in favor of MMC. For instance, recurrence rates as low as 10%-17% have been reported in MMC-treated patients, compared to 40%-50% in controls [14]. Both clinical and animal models consistently confirm its role in attenuating fibrosis by inhibiting fibroblast proliferation and collagen synthesis [15].

Tamoxifen, a selective estrogen receptor modulator, has also shown promising antifibrotic potential beyond its traditional oncologic role. Mechanistically, it inhibits TGF-β signaling [16] and protein kinase C [17], thereby suppressing fibroblast activity and collagen deposition [18]. In a randomized clinical trial, oral tamoxifen (10 mg twice daily for six months) significantly reduced lower urinary tract symptom scores, improved peak urinary flow, and decreased stricture width compared to controls following IOU [19]. Although clinical data remain limited, these findings suggest that tamoxifen could be a valuable non-invasive antifibrotic agent.

Despite encouraging evidence for both agents individually, no head-to-head randomized controlled trials have yet compared tamoxifen and MMC in the prevention of recurrent urethral stricture. Addressing this gap is essential for establishing the most effective adjuvant strategy in clinical practice. Therefore, the present randomized controlled trial aimed to compare the efficacy of oral tamoxifen and intralesional MMC in preventing recurrence and improving functional outcomes following IOU for anterior urethral stricture. It was hypothesized that tamoxifen, owing to its antifibrotic and anti-inflammatory properties, would produce greater improvement in urinary symptoms and flow rates without increasing recurrence risk compared to MMC. The primary objective was to compare changes in the International Prostate Symptom Score (IPSS) [19] at three and six months postoperatively, while secondary objectives included evaluating differences in maximum urinary flow rate (Qmax), stricture length and width, recurrence rate, and recurrence-free period between the two groups. The primary outcome measure was the change in IPSS from baseline to six months, with Qmax, stricture dimensions, recurrence rate, and recurrence-free period serving as secondary outcome measures.

## Materials and methods

Study design and setting

**Figure 1:**
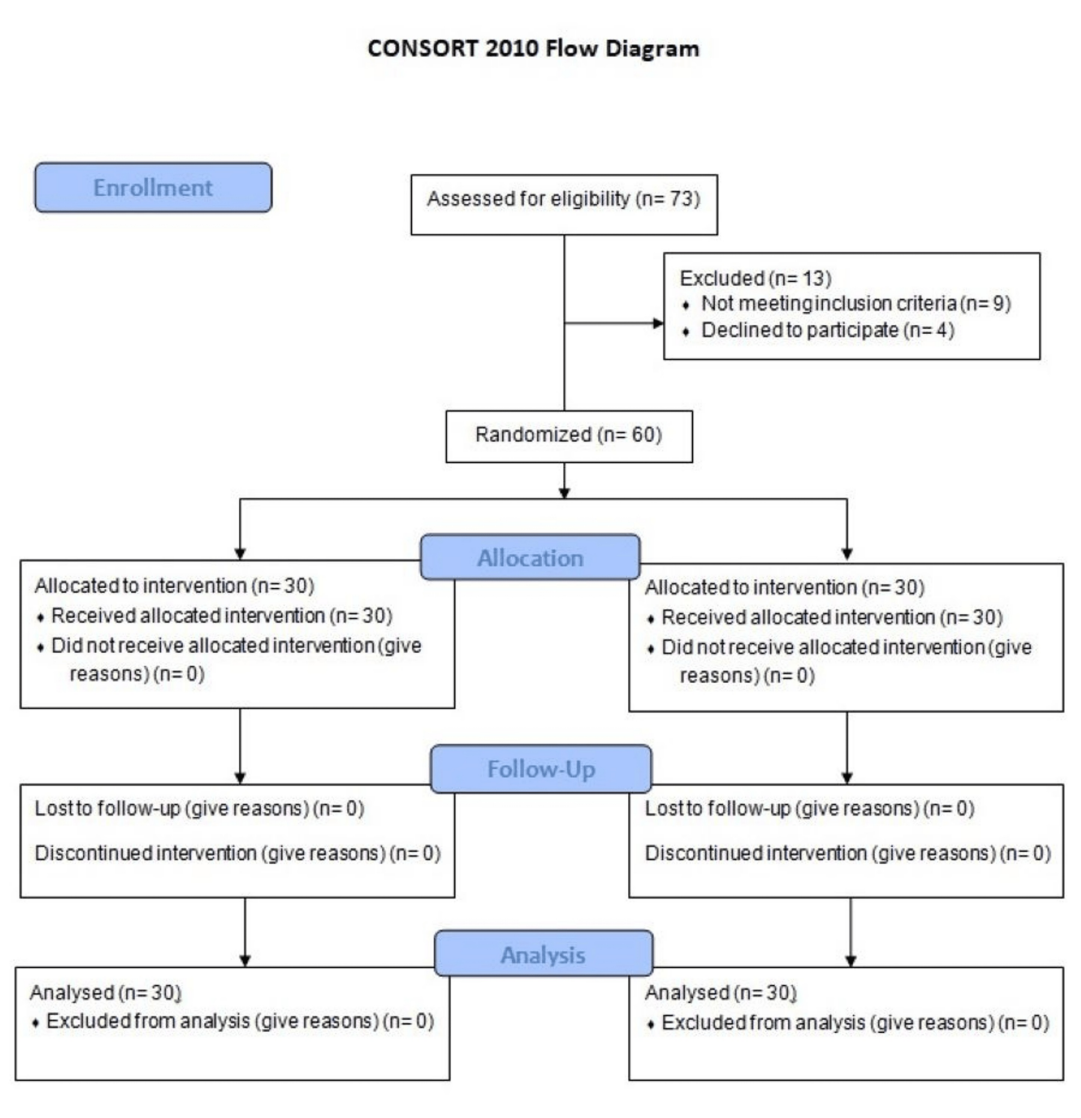
CONSORT Flow Diagram

This was a prospective, randomized clinical study conducted to compare the effectiveness of tamoxifen and MMC as adjuvant therapies in preventing recurrence of urethral stricture following IOU. The study was performed in the Department of Urology at a tertiary care center over the period from January 16, 2025, to July 17, 2025. It was approved by the Institutional Ethical Review Committee (Approval No. 547-03/01/Chairman/R&E/Committee/IKD, dated January 16, 2024) and registered with the Chinese Clinical Trial Registry under registration number ChiCTR2500109869. The trial followed CONSORT guidelines (Figure 1).

Patient selection

The sample size was calculated using prior studies comparing adjuvant agents for preventing stricture recurrence after IOU. Assuming a recurrence rate reduction from 45% to 20%, a power of 80%, and a significance level of 5%, the minimum sample required was 27 per group. To accommodate potential losses to follow-up, 30 participants were enrolled in each group, yielding a total of 60 patients. Male patients aged 18 years or older with primary or recurrent anterior urethral stricture, adequate spongiosal tissue, no extensive fibrosis ≤2 cm in length, Qmax ≤15 mL/s, and IPSS ≥12 were eligible. Exclusion criteria included posterior urethral stricture, multiple recurrent urethral strictures, history of urethral surgery other than IOU, active urinary tract infection, urethral malignancy, uncontrolled comorbidities (e.g., diabetes mellitus and coagulopathy), and contraindication to tamoxifen or MMC. The majority of enrolled cases had bulbar involvement, although penile urethral strictures were also included when meeting the above criteria.

Randomization and grouping

Patients were randomized in a 1:1 ratio to receive tamoxifen or MMC using a computer-generated random sequence, with allocation concealment in sealed opaque envelopes. Surgeons were aware of treatment allocation due to the nature of the intervention, but patients and outcome assessors were blinded. All patients underwent standard IOU under spinal anesthesia. After completion of the IOU, the assigned adjuvant treatment was applied. Group A received oral tamoxifen 10 mg twice daily, while Group B received intralesional MMC (10 mg/mL) at the site of urethrotomy. A urethral catheter was inserted for five to seven days postoperatively in both groups.

Surgical procedure

All patients underwent IOU under spinal anesthesia using a cold knife urethrotome. Strictures were incised at the 12 o’clock position until the urethral lumen was adequately restored and petechial hemorrhages from the spongy tissue were observed, indicating adequate restoration of the urethral lumen. A 16 Fr Foley catheter was inserted postoperatively and kept for five to seven days to permit mucosal healing and minimize the risk of re-adhesion at the urethrotomy site, in accordance with established postoperative protocols.

Outcome measures

The primary outcome of the study was the IPSS, used to assess lower urinary tract symptoms and functional improvement following IOU. Secondary outcomes included Qmax, measured by uroflowmetry, and stricture length and width, assessed through perineal ultrasonography - a non-invasive alternative to retrograde urethrography for anterior urethral strictures. In addition, the recurrence-free period was evaluated as an analytical measure. It was defined as the time interval between the date of IOU and the first documented recurrence of urethral stricture, determined by symptomatic worsening (increase in IPSS), reduction in Qmax (<10 mL/s), or evidence of re-narrowing on imaging. Patients without recurrence at six months were censored at their last follow-up visit.

Statistical analysis

Data were analyzed using IBM SPSS Statistics for Windows, Version 26 (Released 2018; IBM Corp., Armonk, NY, USA). Normality was tested using the Shapiro-Wilk test. Continuous variables were expressed as mean ± standard deviation and compared using the independent-samples t-test or Mann-Whitney U test. Categorical variables were compared using Chi-square or Fisher’s exact tests. The recurrence-free period was assessed using Kaplan-Meier analysis and compared with the log-rank test. A p-value <0.05 was considered statistically significant.

## Results

Baseline characteristics

A total of 60 patients meeting the inclusion criteria were enrolled and randomized equally into the tamoxifen group (n = 30) and the MMC group (n = 30). The mean age was 45.13 ± 7.86 years in the tamoxifen group and 46.10 ± 8.02 years in the MMC group (p > 0.05). Baseline stricture length, stricture width, IPSS, and Qmax were comparable between groups (Table 1). No statistically significant differences were observed, confirming that randomization resulted in balanced baseline characteristics.

**Table 1:** Baseline Characteristics of Study Participants Values are presented as mean ± SD. Between-group comparisons were performed using the independent-samples t-test. A p value <0.05 was considered statistically significant.

Variable	Tamoxifen (n = 30)	Mitomycin C (n = 30)	p-value
Age (years)	45.13 ± 7.86	46.10 ± 8.02	0.64
Stricture length (cm)	1.15 ± 0.38	1.28 ± 0.40	0.15
Stricture width (mm)	3.49 ± 0.89	3.43 ± 0.75	0.77
IPSS	22.17 ± 4.56	21.57 ± 4.51	0.64
Qmax (mL/s)	7.51 ± 1.44	7.45 ± 1.29	0.88

Primary outcome - IPSS

**Figure 2:**
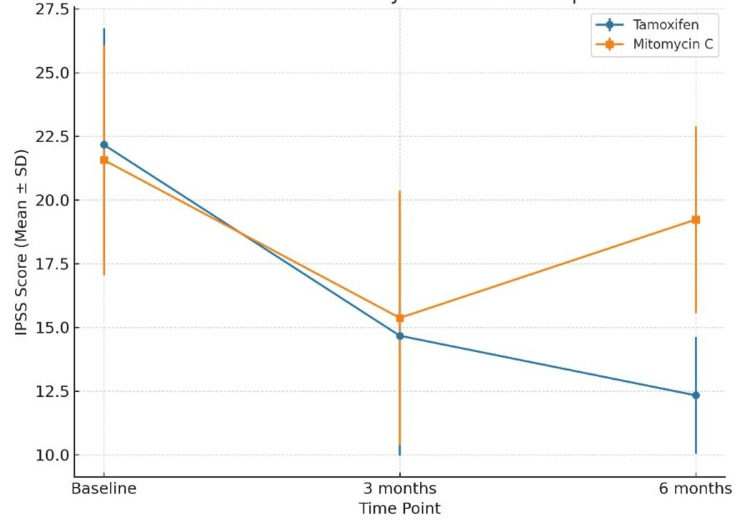
Changes in IPSS (Mean ± SD) From Baseline to Six Months in the Tamoxifen and Mitomycin C Groups Between-group comparisons were performed using the independent-samples t-test. A p-value <0.05 was considered statistically significant.

At baseline, mean IPSS was 22.17 ± 4.56 in the tamoxifen group and 21.57 ± 4.51 in the MMC group (p > 0.05). At three months, scores decreased to 14.67 ± 4.71 and 15.37 ± 4.99, respectively (p = 0.58). At six months, tamoxifen patients had a mean IPSS of 12.33 ± 2.29 compared to 19.23 ± 3.66 in the MMC group, demonstrating a statistically significant improvement with tamoxifen (t = -8.742, p < 0.001), indicating greater symptom relief in the tamoxifen group (Figure 2).

Secondary outcome - Qmax

**Figure 3:**
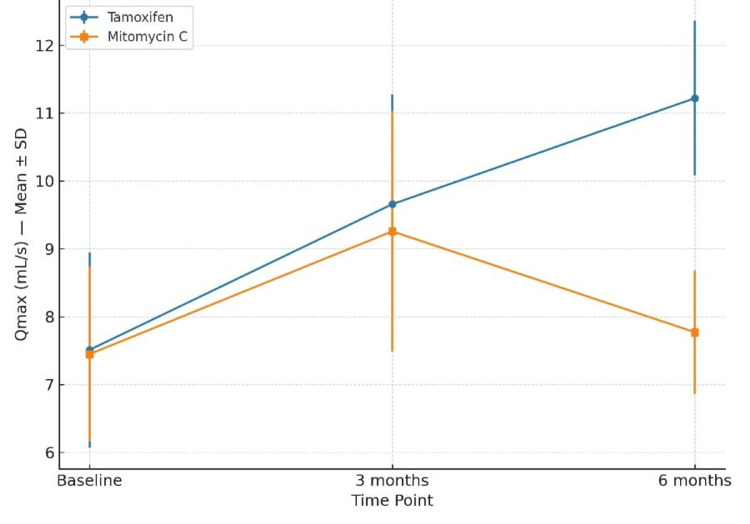
Changes in Qmax (Mean ± SD) From Baseline to Six Months in the Tamoxifen and Mitomycin C Groups Between-group comparisons were performed using the independent-samples t-test. A p-value <0.05 was considered statistically significant.

Baseline Qmax was 7.51 ± 1.44 mL/s in the tamoxifen group and 7.45 ± 1.29 mL/s in the MMC group (p > 0.05). At three months, Qmax improved to 9.66 ± 1.62 and 9.26 ± 1.77, respectively (p = 0.36). At six months, tamoxifen achieved a mean Qmax of 11.22 ± 1.14, while MMC declined to 7.77 ± 0.91 (t = 12.983, p < 0.001) (Figure 3).

Stricture dimensions

**Figure 4:**
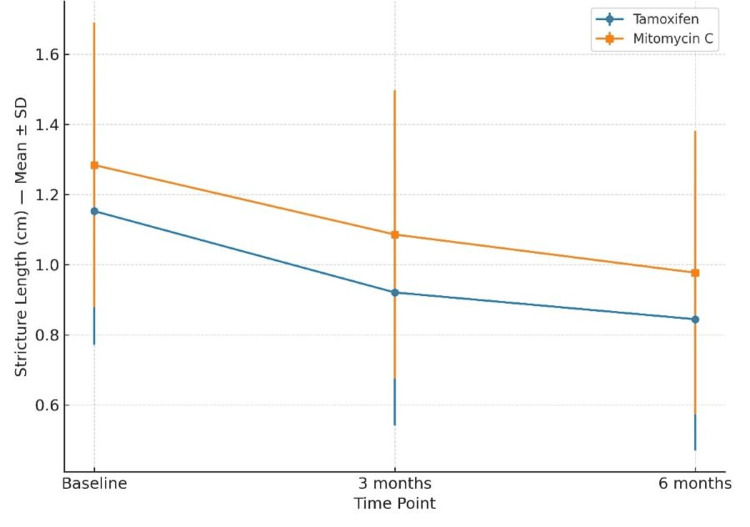
Changes in Stricture Length (Mean ± SD) From Baseline to Six Months in the Tamoxifen and Mitomycin C Groups Between-group comparisons were performed using the independent-samples t-test. A p-value <0.05 was considered statistically significant.

At baseline, mean stricture length was 1.15 ± 0.38 cm in the tamoxifen group and 1.28 ± 0.40 cm in the MMC group (p > 0.05). At three and six months, there were no statistically significant differences between groups (p = 0.119 at three months; p = 0.191 at six months) (Figure 4).

**Figure 5:**
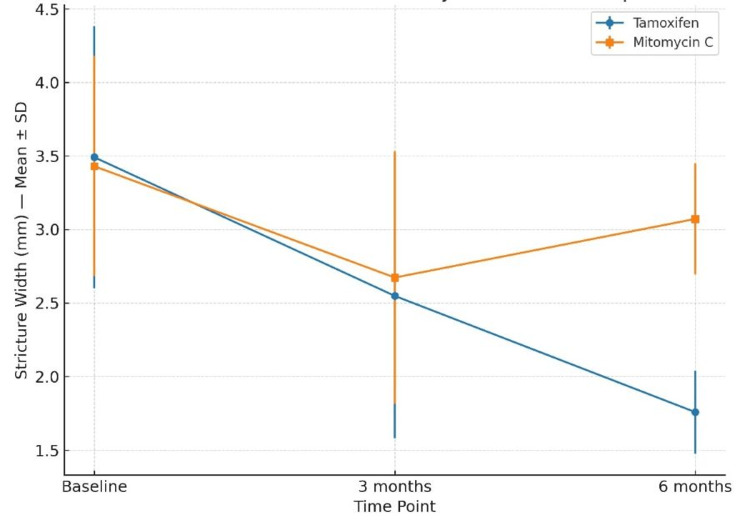
Changes in Stricture Width (Mean ± SD) From Baseline to Six Months in the Tamoxifen and Mitomycin C Groups. Between-group comparisons were performed using the independent-samples t-test. A p-value <0.05 was considered statistically significant.

At baseline, mean stricture width was 3.49 ± 0.89 mm in the tamoxifen group and 3.43 ± 0.75 mm in the MMC group (p > 0.05). At six months, tamoxifen had a significantly narrower mean stricture width (1.76 ± 0.28 mm) compared to MMC (3.07 ± 0.38 mm; t = -15.233, p < 0.001) (Figure 5). The longitudinal changes in IPSS, Qmax, stricture length, and stricture width over the follow-up period are summarized in Table 2.

**Table 2:** Changes in IPSS, Qmax, Stricture Length, and Stricture Width Over Follow-Up Values are presented as mean ± SD. Between-group comparisons were performed using the independent-samples t-test. A p value <0.05 was considered statistically significant.

Outcome	Time Point	Tamoxifen (Mean ± SD)	Mitomycin C (Mean ± SD)	p-value
IPSS	3 months	14.67 ± 4.71	15.37 ± 4.99	0.58
	6 months	12.33 ± 2.29	19.23 ± 3.66	<0.001
Qmax (mL/s)	3 months	9.66 ± 1.62	9.26 ± 1.77	0.36
	6 months	11.22 ± 1.14	7.77 ± 0.91	<0.001
Stricture length (cm)	3 months	0.92 ± 0.38	1.09 ± 0.41	0.12
	6 months	0.84 ± 0.37	0.98 ± 0.40	0.19
Stricture width (mm)	3 months	2.55 ± 0.97	2.67 ± 0.86	0.61
	6 months	1.76 ± 0.28	3.07 ± 0.38	<0.001

Recurrence rate

Recurrence was observed in 10/30 (33.3%) patients in the tamoxifen group and 11/30 (36.7%) in the MMC group. The difference was not statistically significant (p = 0.787) (Table 3).

**Table 3:** Recurrence Rates and Survival Analysis * denotes log-rank test. Categorical variables were compared using the Chi-square test. Recurrence-free survival was assessed using Kaplan-Meier analysis and compared with the log-rank test. A p-value <0.05 was considered statistically significant.

Variable	Tamoxifen (n = 30)	Mitomycin C (n = 30)	p-value	Odds Ratio (95% CI)
Recurrence rate, n (%)	10 (33.3)	11 (36.7)	0.787	1.158 (0.400-3.348)
Mean recurrence-free period (days)	142.7 (95% CI: 120.06-165.33)	133.2 (95% CI: 109.52-156.88)	0.748*	-

Kaplan-Meier survival analysis

**Figure 6:**
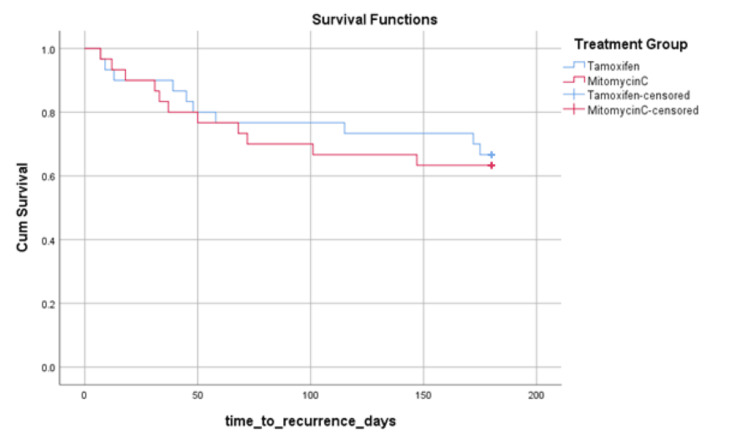
Kaplan-Meier Survival Curves Showing Recurrence-Free Survival in Tamoxifen and Mitomycin C Groups Differences between groups were compared using the log-rank test. A p-value <0.05 was considered statistically significant.

Kaplan-Meier analysis for recurrence-free period showed a mean recurrence-free duration of 142.7 days (95% CI: 120.06-165.33) for tamoxifen and 133.2 days (95% CI: 109.52-156.88) for MMC (log-rank p > 0.05) (Figure 6).

## Discussion

This randomized controlled trial compared the effectiveness of oral tamoxifen versus intralesional MMC in preventing recurrent anterior urethral stricture, predominantly bulbar, following IOU. Both adjuvant agents demonstrated improvement in urinary symptoms and flow rates in the early postoperative period. However, at six months, tamoxifen was associated with significantly greater improvement in the IPSS, Qmax, and stricture width compared with MMC, while recurrence rates were similar between the two groups.

The reduction in IPSS and improvement in Qmax observed in both treatment arms at three months are consistent with the expected short-term benefits of IOU and adjuvant therapy. By six months, patients in the tamoxifen group maintained significantly greater improvement in IPSS (12.33 ± 2.29 vs. 19.23 ± 3.66; p < 0.001) and Qmax (11.22 ± 1.14 vs. 7.77 ± 0.91 mL/s; p < 0.001) compared with the MMC group. This suggests that tamoxifen may exert a more sustained anti-fibrotic effect, potentially translating to prolonged symptom relief despite similar anatomical recurrence rates.

Our results are in line with previous reports assessing MMC as an adjuvant following IOU. Al-Falah et al. [20] evaluated anterior urethral strictures and found that patients receiving MMC had a substantially lower recurrence rate compared to controls, although improvements in maximum urinary flow and stricture length were not statistically significant. Similarly, Xu et al. [21] conducted a meta-analysis of nine trials, including 550 participants, showing that MMC significantly reduced the recurrence of short anterior urethral strictures, especially when follow-up extended beyond one year. Jacobs et al. [22] reported that MMC, alone or combined with hyaluronic acid and carboxymethylcellulose, reduced stricture recurrence, but the benefit decreased over longer follow-up periods. In another study, Moradi et al. [23] observed a marked reduction in recurrence with MMC compared to control patients, with minimal associated complications. Collectively, these findings suggest that MMC may effectively lower early recurrence after IOU for short anterior strictures, though its long-term benefits remain variable, highlighting the need for further well-powered, multicenter trials.

Tamoxifen has been less extensively studied in urethral stricture disease, but its antifibrotic activity appears to involve downregulation of TGF-β and modulation of collagen metabolism, as demonstrated in renal fibrosis models - where it reduced collagen I/III, fibronectin, and TGF-β1 expression, and inhibited fibroblast proliferation [24]. Similar findings were observed in both rat and human kidney fibrosis models, with decreased collagen levels [25]. Although no direct animal model of urethral stricture has been conducted, tamoxifen did significantly affect urethral tissue morphology in treated rats [26]. Clinically, our results align with a randomized trial by El-Shazly et al. [19], who reported that patients receiving tamoxifen post-IOU demonstrated significantly lower IPSS scores and higher Qmax compared to controls. They also reported a smaller stricture width in the tamoxifen group on perineal ultrasonography.

The lack of a statistically significant difference in recurrence rates between the two groups may be attributed to our follow-up duration of only six months. Urethral stricture recurrence often occurs beyond the first postoperative year, and longer-term data are necessary to determine whether the symptomatic advantage with tamoxifen translates into a lower long-term recurrence rate [22,27]. Additionally, as in other urethral stricture research [28], our trial was powered to detect differences in IPSS, not recurrence rates, which may explain the absence of statistical significance for this outcome.

From a clinical perspective, the superior improvement in urinary function observed with tamoxifen is noteworthy. Even if anatomical recurrence is not significantly reduced, improved Qmax and IPSS may positively impact quality of life, patient satisfaction, and the need for re-intervention. This study has several strengths, including its randomized design, balanced baseline characteristics, use of a standardized IOU technique, and comprehensive functional and anatomical outcome assessment. However, some limitations must be acknowledged. First, the follow-up period was relatively short, potentially underestimating late recurrences. Second, the single-center design may limit generalizability. Third, blinding of the surgical team was not possible due to the nature of the intervention, which could introduce performance bias. Lastly, the sample size was modest and primarily included smaller urethral strictures (≤2 cm), which may overestimate functional improvements and underpower the detection of differences in recurrence rates.

Future research should focus on large, multicenter trials with longer follow-up to confirm these findings and assess the durability of the functional benefits observed with tamoxifen. Dose-response studies may help optimize application protocols. Additionally, combining tamoxifen with other antifibrotic agents or mechanical interventions may offer synergistic benefits in recurrence prevention.

## Conclusions

In this randomized controlled trial, both oral tamoxifen and intralesional MMC demonstrated short-term improvements in urinary symptoms and flow rates following IOU for anterior urethral strictures. Oral tamoxifen provided significantly greater functional improvement and reduction in stricture width at six months, despite similar recurrence rates between groups. These findings should be interpreted with caution, as the study included a modest sample size and predominantly smaller urethral strictures (≤2 cm), which may limit generalizability. Nonetheless, tamoxifen appears to be a promising, low-cost, and easily administered adjuvant therapy. Larger, multicenter trials with longer follow-up and recurrence as the primary endpoint are warranted to confirm these results and determine their efficacy in longer or more complex strictures.
